# Local site differences in survival and parasitism of periwinkles (*Littorina sitkana* Philippi, 1846)

**DOI:** 10.1002/ece3.2708

**Published:** 2017-01-18

**Authors:** Mónica Ayala‐Díaz, Jean M. L. Richardson, Bradley R. Anholt

**Affiliations:** ^1^ Bamfield Marine Sciences CentreBamfieldBCCanada; ^2^ Department of BiologyUniversity of VictoriaVictoriaBCCanada

**Keywords:** Barkley Sound, MARK, Mark‐Release‐Recapture, parasites, snail ecology, WinBUGS

## Abstract

The periwinkle, *Littorina sitkana*, is found throughout the intertidal zone, often in isolated subpopulations. The majority of trematode parasites use snails as intermediate hosts, and decreased survivorship is often observed in snails infected with trematodes. Sampling *L. sitkana* from four sites in Barkley Sound, British Columbia, Canada, we test the effects of parasitic infection on snail survival using maximum likelihood and Bayesian approaches using the software MARK and WinBUGS. We found that survival of periwinkles and trematode community composition differed among sites, but survival and trematode prevalence were uncorrelated. WinBUGS performed better than MARK in two ways: (1) by allowing the use of information on known mortality, thus preventing survival overestimation; and (2) by giving more stable estimates while testing the effect of body size on snail survival. Our results suggest that snail survival depends heavily on local environmental factors that may vary greatly within a small geographical region. These findings are important because the majority of experimental studies on survival are done on snails from a single location.

## 1 Introduction

Survival of organisms is likely to vary among sites (Einum & Nislow, 2005; Price, Eskew, Cecala, Browne, & Dorcas, 2012; Reznick & Bryant, 2007; Smith, Finch, & Stoleson, 2014). Variation in survival rates among sites may be related to differences in human impact (Price et al., 2012), vegetation presence and composition (Segura, Masson, & Gantchoff, 2012; Smith et al., 2014), population densities (Einum & Nislow, 2005; Nail, Stenoien, & Oberhauser, 2015), microclimate (Bertrand & Wilson, 1996), predator and parasite presence (Fredensborg, Mouritsen, & Poulin, 2005; Reznick & Bryant, 2007), among numerous other abiotic or biotic factors.

Snails experience substantial parasitism rates, as most of the known trematode parasites worldwide use snails as their first intermediate host (Esch, Curtis, & Barger, 2001). Trematode parasites can decrease the survival of snail intermediate hosts in a variety of ways: some trematode parasites manipulate snail behavior, increasing predation risk on snail hosts along with parasite transmission to the next host (Johnson, Lunde, Haight, Bowerman, & Blaustein, 2001; Thomas & Poulin, 1998; Thomas, Poulin, & Brodeur, 2010); trematodes can decrease snail survival in oxygen and nutrient‐limited habitats (Fredensborg et al., 2005; Sousa & Gleason, 1989); and trematodes can also decrease snail survival rates due to strong immune responses from the host to parasitic infection, as well as through tissue damage occurring while the parasite feeds, or during cercarial release (Minchella, 1985; Sorensen & Minchella, 2001). As a result, it seems likely that parasite populations in a local habitat will affect snail survival. Trematode communities vary among populations (Faltynkova, Valtonen, & Karvonen, 2008; Galaktionov & Bustnes, 1999; Granovitch, Sergievski, & Sokolova, 2000; Hechinger & Lafferty, 2005; Thieltges et al., 2009) and variation in trematode species distribution and/or prevalence leads to differences in survivorship among sites (Granovitch & Maximovich, 2013).

Two methods are widely used in ecology for measuring survival of intertidal snails in the field: tethering (Behrens Yamada & Boulding, 1996; Rochette & Dill, 2000) and Mark‐Release‐Recapture (MRR) (Kovach & Tallmon, 2010; López‐Rocha & Naegel, 2007). The tethering method has the advantage of preventing dead snails from being swept away by wave action, but its set‐up is time‐consuming. This limits the number of animals that can be tested when working in intertidal zones, as experimental set‐up must take place during the short time available between high tides (Mónica Ayala‐Díaz, personal observation). The addition of predator exclusion cages to tethering experiments is helpful to explain the differences in survival of tethered animals, but cage size (i.e., surface area) and the time cages and tethered snails can remain in place are restricted due to wave exposure. In addition, small, but biologically important, differences in survival may not be detectable using short time periods (Mónica Ayala‐Díaz, personal observation).

Mark‐Release‐Recapture techniques have two logistic advantages over tethering methods for experimentation in the intertidal zone: (1) snail marking can be done in the laboratory, avoiding time restrictions imposed by changing tides while in the field, and (2) time between recapture occasions can be longer, making differences in survival easier to detect. However, such logistic advantages are counter‐balanced by uncertainty regarding the cause of differential survivorship and the need for more sophisticated models to analyze the data. Emigration from sites during an MRR study can confound estimates of survival. However, intertidal snails stay close to their settlement site (Bates & Hicks, 2005), and thus marked snails are unlikely to leave the study area, allowing us to assume emigration rates of zero. This reduces the number of unknown parameters during estimation, simplifying survival data analysis and increasing recapture probabilities. Two commonly used methods for analysis of MRR data are as follows: (1) general maximum likelihood (ML) methods and (2) Bayesian inference methods. The software MARK (White & Burnham, 1999) uses general ML techniques for analysis of MRR data and is widely used by researchers. In this method, parameters are treated as unknown fixed constants that are estimated by maximizing the joint likelihood function of the data. Uncertainty around parameter estimates is estimated based on the frequency of parameter estimates from hypothetical replicates of the data. Bayesian inference methods treat parameters as random variables and uncertainty is estimated as the conditional posterior probability distribution of the parameter (Kéry, 2010). The freely available software WinBUGS allows flexible analysis of data sets using Bayesian inference (Lunn, Thomas, Best, & Spiegelhalter, 2000).

Here, we estimate survival of *L. sitkana* for each of four sites using MRR methods over 8 months and test the hypothesis that survival is related to trematode prevalence. We use traditional ML estimation using MARK (White & Burnham, 1999) and compare these to estimates obtained through Bayesian inference using the program WinBUGS (Lunn et al., 2000).

## 2 Methods

We collected *L. sitkana* (>7 mm shell height, measured from the bottom of the shell's outer lip to the shell's apex) by searching the rocky intertidal zone of four sites located on the West Coast of Vancouver Island, Canada. Two sites were on the mainland: Prasiola Point (125° 10′ 4.42″W, 48° 49′ 1.14″N) and Nudibranch Point (125° 10′ 29.72″W, 48° 48′ 53.73″N), separated by 550 m from each other. Mainland sites are located in a sheltered zone and thus have low wave exposure. Two islet sites, separated from mainland sites by 6 km, were also used: Ross Islet (125° 9′ 43.18″W, 48° 52′ 26.13″N) and Wizard Islet (125° 9′ 35.14″W, 48° 51′ 29.25″N), separated by 1.78 km from each other. Islets are exposed to high wave action. After collection, snails were transported within an hour to the laboratory at the Bamfield Marine Sciences Centre (BMSC), where they were kept in sea tables with constant sea water flow (sea water was pumped from 20 m deep in the inlet and was approximately 10°C). *Ulva intestinalis* Linnaeus 1753 collected from nearby field sites was provided as a food source ad libitum. Protocol and procedures for this study were reviewed and approved by the Animal Care Committee at BMSC.

### 2.1 Snail survival

From March to October 2012, we conducted an intensive MRR experiment in the field. To estimate survival using an MRR approach, individuals must receive, at a minimum, a cohort mark identifying time of release, and an encounter history must be created for each individual to summarize each recapture event, assigning a value of “1” if they were captured alive and a value of “0” otherwise (Cooch & White, 2011). This allows the estimation of temporally variable survival rates independent of recapture probability. For each of the four sites, we marked eight cohorts (once every 20 days) of snails with individual tags and had nine recapture occasions. We collected a new cohort of snails 2 days before each recapture occasion and tagged them in the laboratory. On each recapture occasion, we released newly marked snails immediately after we finished the survival census of the previously released cohorts. All cohorts of periwinkles were released at their site, in clumps, into three to four tide pools found within an area of approximately 2 m^2^. Tide pool diameters ranged from approximately 60 to 120 cm.

Each released cohort had between 52 and 300 tagged snails. We marked a total of 8,772 snails by attaching uniquely numbered all‐weather paper tags to the shell of each snail using super glue, and then covering the tags with clear nail polish. The use of unique individual marks allows the incorporation of individual covariates (e.g., body size) into the analysis. Survival can be underestimated when lost marks are interpreted as lost or dead individuals. Thus, the use of a second marking method (a principal mark and an accessory one) can limit the risk of survival underestimation due to mark loss (Juillet, Choquet, Gauthier, & Pradel, 2010). To decrease the risk of underestimating survival due to tag loss, we applied a secondary mark of colored nail polish on the outer apertural rim of the shell of each snail, using a different color for each cohort.

Twenty days after release, we returned to each site and searched thoroughly for marked snails along visual transects parallel to the waterline and starting at the furthest place from the release point where we could find marked individuals. We considered a recapture occasion as complete, once no more snails were found in a radius of approximately 6 m from the release point. Each time we spotted a marked snail, we recorded its tag number and immediately replaced the snail at the location in which it was found. We noted empty marked shells as dead recoveries (Juillet et al., 2010). Marked snails with damaged or missing numbered tags but with the colored cohort mark were removed from the study site noting the date of resighting and their cohort number. Field collections were approved by the Department of Fisheries and Oceans Canada (DFO) (permit XR 61 2011) and the Huu‐ay‐aht First Nations office for access to protected lands.

For each cohort at each site, we also kept a sample of snails (*n* = 29 ± SE 0.06) for dissection to estimate trematode prevalence. These snails were held under laboratory conditions, and provided an estimate of survival in the absence of predation. We compared the proportion of snails that survived in the laboratory with survival rates estimated from the MRR experiment conducted in the field.

### 2.2 Trematode infection status

We dissected samples of 29 ± 0.06 (mean ± SE) snails collected from the four sites every month from March to September 2012 (*N* = 922 snails) for identification of trematode infection. To assess trematode community composition of periwinkles correctly, and to ensure that all trematodes present were detected, we crushed the shell of each snail and thoroughly examined the contents of digestive and reproductive glands using an inverted microscope to look through the entire sample. All trematodes were identified based on morphology to the lowest taxonomic level possible using identification keys by Ching (1963, 1991), Gorbushin and Shaposhnikova (2002), James (1968), Saville, Galaktionov, Inwin, and Malkova (1997), and Yamaguti (1975). We measured the shell height of the snails as for the MRR study.

### 2.3 Statistical analysis

We found 13 live snails with colored mark on shell apex, but without numbered tags; these could be misinterpreted as dead snails during the analysis, leading to survival underestimation. To prevent this, prior to analyzing MRR data we randomly removed 13 capture histories from the respective cohort containing only zeroes after the recapture occasion in which we found a snail without a numbered tag. We also removed the capture histories of dead individuals (*n* = 483) to improve estimate precision (Cooch & White, 2011) although this may lead to a small overestimate of survival. We analyzed live recapture data of 8,276 snails using the Cormack–Jolly–Seber (CJS) models in MARK (White & Burnham, 1999). MARK allows the use of individual covariates while estimating survival, but residuals from our data including snail size as a covariate were highly overdispersed, suggesting estimate unreliability; thus, we show results from the simplest CJS model (Φ.*p*. no time‐dependence) without covariates here. We compared these results to survival estimates obtained through Bayesian inference using WinBUGS (Lunn et al., 2000) by fitting a state‐space CJS model to analyze live recaptures, as described by Royle (2008). In this model, we were able to include snail shell height as an individual covariate to assess the effect of body size on survival and recapture rate (Royle, 2008). Further, in WinBUGS, we were able to include information on known deaths, preventing survival overestimation and increasing the number of snail capture histories analyzed to 8,759. We ran state‐space models from within R using the R2WinBUGS package (Sturtz, Ligges, & Gelman, 2005), with 300,000 iterations of three chains, a burn‐in of 10,000 and 100 as thinning. We analyzed model outputs using the R package Coda (Plummer, Best, Cowles, & Vines, 2006). Uninformative priors were used for all parameters to avoid biased estimates. We analyzed data from each site separately for both MARK and WinBUGS in order to make models computationally practical.

For each site, we estimated trematode species richness as the number of trematode species at the site, trematode presence as the number of snails that had at least one species of trematode, and trematode prevalence as the percentage of snails infected with a particular species of trematode. We analyzed differences in trematode presence with a Pearson chi‐square (χ^2^) test of independence in R (R Development Core Team, 2010). Species’ prevalence data were analyzed with generalized linear models in R. For each trematode species, we used trematode species’ presence as the response variable, coded as a matrix of number of successes (if trematode species was present in a snail) and failures (if trematode species was absent in a snail) and site as the explanatory variable. We used planned contrasts to compare sites for each trematode species. Data were analyzed using the glm() function in R, specifying binomial distribution and logit link function. We analyzed snail size data with the aov() function in R, transforming shell height data to their natural logarithm to improve normality.

Using information of trematode presence per cohort and capture histories from the four study sites within program MARK, we tested several biological hypotheses to determine if snail survival is affected by trematode presence. We compared models representing different hypotheses based on their Akaike information criterion (AIC) scores. We also tested the hypothesis that survival estimates were correlated with trematode presence using the function cor.test() in R. We analyzed data separately by site to get more reliable correlation coefficients.

## 3 Results

### 3.1 Snail survival

**Figure 1:**
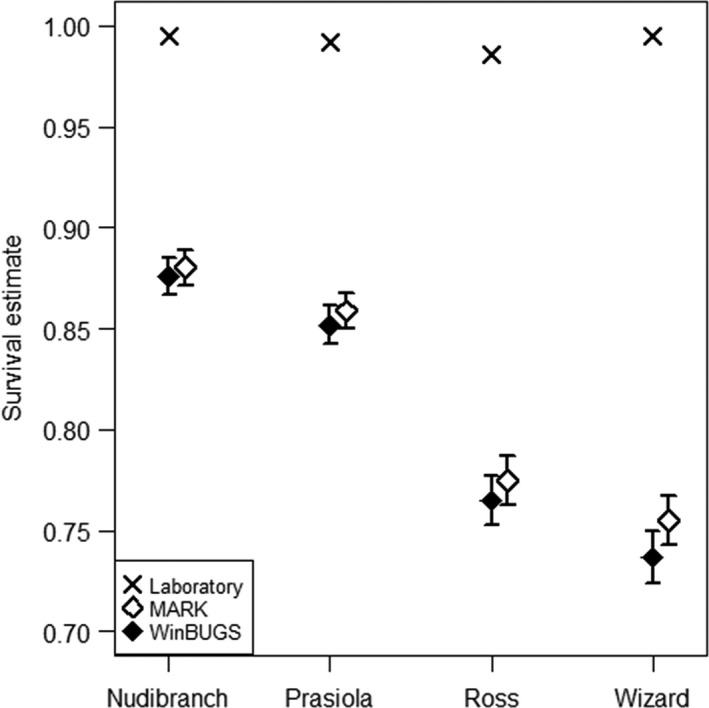
Mean survival estimates ± Credible (CRI) or Confidence Intervals (CI) of *Littorina sitkana* from each collection site. Estimates presented as proportion of periwinkle populations surviving for a period of 181 days in the field. Bayesian estimates and CRIs calculated using WinBUGS; Maximum likelihood estimates and CIs calculated with MARK. For comparison, mean survival of snails from each site that were housed in the laboratory is also plotted

Mean survival estimates for snails kept in the laboratory without predation ranged from 0.990 to 0.995 for 181 days (total duration of field experiments). Snail survival in the laboratory did not differ among study sites (*F*_3,28_ = 0.812, *p* = .498; Figure 1). Survival estimates obtained from the best‐fit CJS model in the program MARK (Φ.*p*.) were highest for snails at Nudibranch Point, lower at Prasiola Point and lowest at Ross and Wizard islets (Figure 1). Results from the state‐space CJS model using WinBUGS show the same pattern as results from MARK (Figure 1). Larger snails had lower survival at Prasiola Point (slope = −0.137; *t*_(2396)_ = −191.68, *p* < .001), Ross Islet (slope = −0.092; *t*_(2272)_ = −115.45, *p* < .001), and Wizard Islet (slope = −0.107; *t*_(2102)_ = −213.39, *p* < .001), but slightly higher survival at Nudibranch Point (slope = 0.003; *t*_(1981)_ = 3.93, *p* < .001). Although effects of size on periwinkle survival are small, including snail size in the models run in WinBUGS improved model fit.

**Figure 2:**
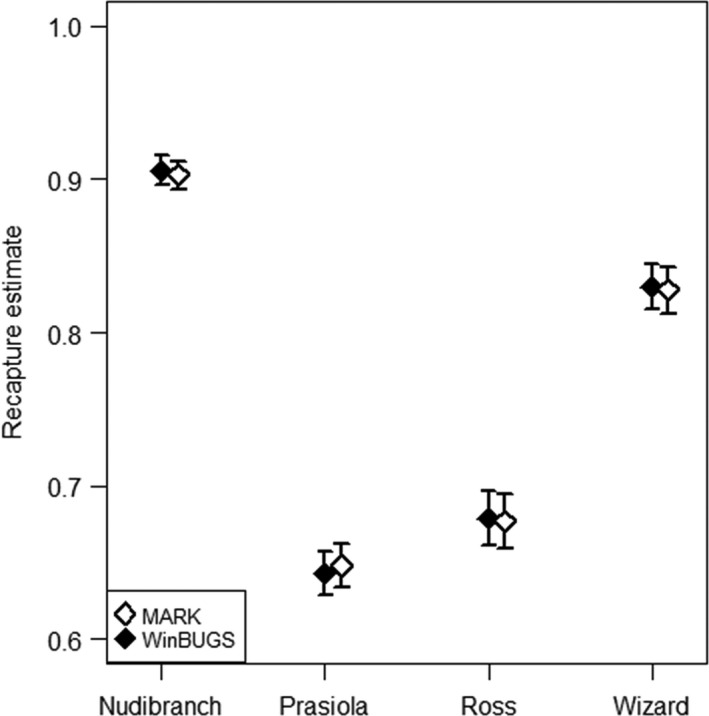
Mean recapture estimates ± Credible (CRI) or Confidence Intervals (CI) of *Littorina sitkana* from each collection site. Estimates presented as proportion of marked periwinkles recaptured after a period of 181 days in the field. Bayesian estimates and CRIs calculated with WinBUGS; maximum likelihood estimates and CIs calculated with MARK

Recapture estimates varied among sites but were almost identical whether estimated by MARK or WinBUGS (Figure 2). Nudibranch Point had the highest recapture estimates, followed by Wizard Islet, while recapture estimates were lowest at Prasiola Point and Ross Islet (Figure 2). Large snails were more likely to be recaptured at all sites (Prasiola: slope = 0.019; *t*_(2396)_ = 30.01, *p* < .001. Nudibranch: slope = 0.272; *t*_(1981)_ = 269.17, *p* < .001. Ross: slope = 0.120; *t*_(2272)_ = 121.75, *p* < .001. Wizard: slope = 0.223; *t*_(2102)_ = 243.55, *p* < .001).

### 3.2 Trematode infection

Trematode presence (the number of snails with at least one trematode) was highest at Wizard Islet (infected snails/*N* = 162/232) and lowest at Ross Islet (38/223), while Nudibranch and Prasiola Points had intermediate trematode presence (81/233 and 86/234, respectively); these site differences were statistically significant ($\chi_{3}^{2}$ = 138.89, *p* < .001).

**Figure 3:**
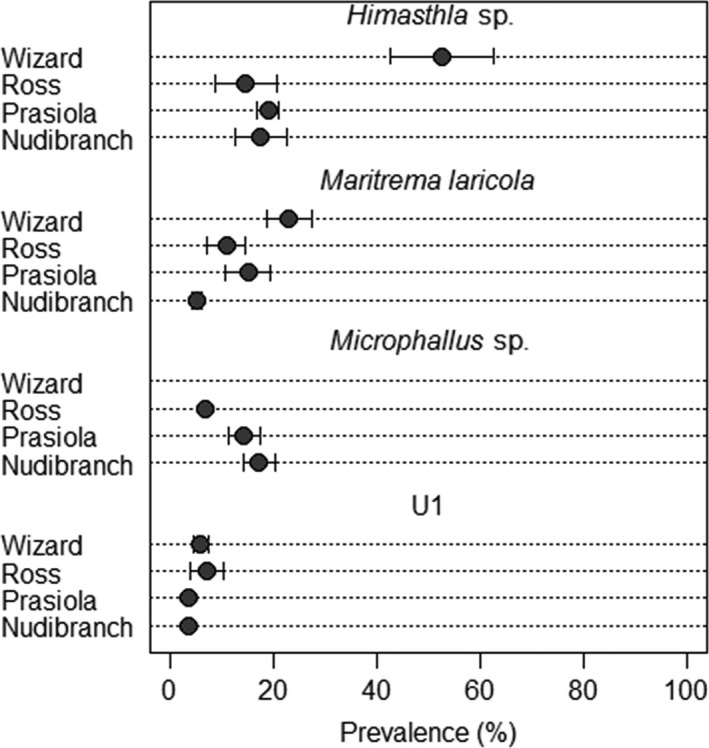
Mean trematode species prevalence in *Littorina sitkana* from each collection site. For several points, error bars do not extend past point symbol

We identified six morphological trematode species in snails (Table 1). *Himasthla* sp. was the most prevalent trematode species at all sites. This species had higher prevalence at Wizard Islet (*z*_26,23_ = 9.48, *p* < .001) when compared with the other three sites. *Himasthla* sp. prevalence did not differ among the other three sites (contrast between Nudibranch and Prasiola Point + Ross Islet: *z*_26,23_ = 0.108, *p* = .914; contrast between Prasiola Point and Ross Islet: *z*_26,23_ = 0.829, *p* = .407) (Figure 3). In snail hosts with *Himasthla* sp., 93% were found as encysted metacercariae.

**Table 1:** Prevalence of trematode species found in *Littorina sitkana* per site

Site	*N*	*Himasthla* sp. (%)	*Maritrema laricola* Ching, 1962 (%)	*Microphallus* sp. (%)	U1 (%)	U2 (%)	U3 (%)
Prasiola Point	234	18.8	9.4	9.4	0.4	0.0	0.0
Nudibranch Point	233	15	2.6	16.7	0.9	0.4	0.4
Ross Islet	223	7.2	8.1	1.8	1.8	0.0	0.0
Wizard Islet	232	53	22.9	0.0	3.5	0.0	0.0

Prevalence of *Maritrema laricola* was highest at Wizard Islet (*z*_22,19_ = 4.32, *p* < .001) and lowest at Nudibranch Point (contrast between Nudibranch and Prasiola Point + Ross Islet: *z*_22,19_ = −2.20, *p* = .028). Prasiola Point and Ross Islet did not differ in prevalence of *Maritrema laricola* (*z*_22,19_ = 1.05, *p* = .296) (Figure 3). This trematode species was always found as sporocysts containing motile cercariae.

*Microphallus* sp. was found as sporocysts containing unencysted metacercariae in all their snail hosts, but was entirely absent from Wizard Islet. This site was thus excluded from the analysis of this species. *Microphallus* sp. had lower prevalence at Ross Islet (*z*_15,13_ = −9.06, *p* < .001), while there was no detectable difference in prevalence between Nudibranch and Prasiola Points (*z*_15,13_ = −0.80, *p* = .426) (Figure 3).

We were unable to identify three trematode species (labeled U1, U2, and U3) that were undeveloped and lacked internal structures. We found U2 and U3 only at Nudibranch Point, with a very low prevalence (Table 1) and these species were excluded from all statistical analyses. Prevalence of U1 did not differ among sites (*z*_8,5_ = −0.55, *p* = .580) (Figure 3). This species was found as sporocysts with non‐motile cercariae.

The best‐fit model in MARK for the effect of trematode presence suggests both snail survival and recapture depend on study site and time but not on trematode presence (Table 2). We tested the hypothesis that trematode presence has long‐term effects on snail survival depending on site, with recapture depending on both site and time. This model was the best fit among hypotheses containing trematode presence, showing the third lowest AIC score of all models tested (Table 2). The hypothesis that trematode presence has immediate effects on snail survival was not well supported by our data; this model ranked 6th (Table 2). Trematode presence does not seem to affect snail recapture probabilities, as the best‐fit model containing long‐term effects of trematode presence in both survival and recapture estimates was ranked 13th and the model containing immediate effects of trematode presence in both survival and recapture was ranked 18th (Table 2).

**Table 2:** Akaike information criterion (AIC) rankings of five most relevant working hypotheses used to determine if trematode presence has an effect on survival and recapture estimates of *Littorina sitkana*. For a full table containing all models compared in this study, refer to Appendix 1

Rank	Model	QAICc	ΔQAICc	No. Parameters	Deviance
1	Φ(site × time) p(site × time)	26159.99	0.00	60	1271.84
3	Φ(site × Trem‐long term) p(site × time)	26283.27	123.28	39	1437.32
6	Φ(site × Trem‐immediate) p(site × time)	26346.69	186.70	40	1498.74
13	Φ(site × Trem‐long term) p(site × Trem‐long term)	26503.55	343.55	16	1703.72
18	Φ(site × Trem‐immediate) p(site × Trem ‐immediate)	26733.53	573.54	16	1933.71

**Figure 4:**
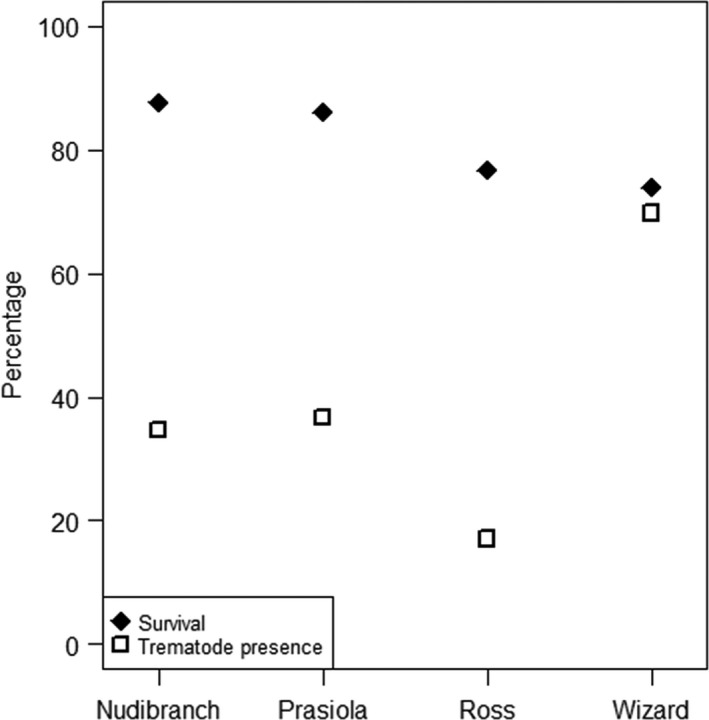
Comparison of mean survival estimates calculated with WinBUGS and trematode presence for each collection site

No significant correlation between survival and trematode presence was observed at any of the study sites (Prasiola: *r* ^2^ = −.001, *p* = .934; Nudibranch: *r* ^2^ = −.122, *p* = .397; Ross: *r* ^2^ = −.024, *p* = .712; Wizard: *r* ^2^ = .195, *p* = .274); trematode presence and snail survivorship were also unrelated among sites (Figure 4).

Snails differed significantly in size among the four sites (*F*_3,912_ = 171.3, *p* < .001); from smallest to largest, means ± SE were as follows: Ross Islet, 12.28 mm ± 0.07; Wizard Islet, 13.36 ± 0.09; Nudibranch Point, 14.16 ± 0.06; and Prasiola Point, 14.45 ± 0.07.

## 4 Discussion

Survival and recapture estimates, trematode prevalence, and size of *L. sitkana* differed significantly among the four study sites. These differences highlight the importance of including more than one location when studying survival. Survival and recapture were high for all sites. Patterns of both survival and recapture estimates from ML and Bayesian models are similar, suggesting estimate reliability. Narrow confidence and credible intervals suggest high estimation precision.

We found a clear difference in survival and recapture between sites located on the mainland and sites on the islets. However, these differences could not be attributed to differences in trematode infection rates among sites. This matches results of O'Dwyer, Kamiya, and Poulin (2014) in New Zealand sites, where recapture rates of infected vs. uninfected periwinkles did not differ significantly. While one of the islets, Wizard, had both the highest trematode infection rate and the lowest survivorship rate (as predicted if trematode parasites lower survival in snail hosts), the other islet site, Ross, had the lowest trematode numbers and survival rates similarly low to Wizard. Further, the two mainland sites had the highest survivorship rates, but intermediate trematode infection rates (Figure 4). In addition, snail survival in the laboratory was similarly high in samples from all sites, contrary to expectations under the hypothesis that snail survival is directly affected by trematode infection (Fredensborg et al., 2005). Differences in trematode prevalence among sites have been previously described and attributed to differences in abundance of definitive hosts among sites (Lambert, Corliss, Sha, & Smalls, 2012; Levakin, Nikolaev, & Galaktionov, 2013). This explanation seems likely for our results, as Wizard Islet has the largest trematode prevalence and the largest population of white‐winged seagulls, while birds are rare on Ross Islet and the mainland sites (Mónica Ayala‐Díaz, personal observation). It thus appears that other factors present in the natural habitat of *L. sitkana* have a larger effect on snail survival than trematode infection or behavioral changes of snail hosts, although a combination of trematode infection and environmental factors is also possible (e.g., a combination of strong wave action and trematode‐induced reduction of periwinkle attachment strength as described in O'Dwyer, Lynch, & Poulin, 2014).

Snail size had a small but significant effect on snail survival and the effect varied among sites, suggesting an interaction is occurring. For all sites but Nudibranch Point, survivorship decreased with increasing size. Given that recapture probability of larger snails was higher than that of smaller snails, lower survival estimates for larger snails seen here are unlikely to be caused by low recapture rates; thus, we are confident that our results show real differences in periwinkle population dynamics among our study sites. It might simply be that larger snails are older and thus, more likely to die during the study than smaller, younger snails. However, other explanations are as plausible. Larger periwinkles are preferred by large predatory crabs (on wave exposed habitats; Behrens Yamada & Boulding, 1996) and fish (on wave protected shores; McCormack, 1982; Rochette & Dill, 2000), and are more likely to be dislodged by wave action due to their larger surface area (Boulding & Van Alstyne, 1993; McCormack, 1982) and because, being unable to fit into protected crevices or between barnacles, they spend more time exposed on rock surfaces (O'Dwyer, Kamiya et al., 2014; Rickards & Boulding, 2015; Silva, Mendonça, Paquete, Barreiras, & Vinagre, 2015). The latter hypothesis combined with smaller mean shell height of *L. sitkana* on islets suggest a plausible explanation for lower survival of *L. sitkana* at Ross and Wizard islets; if larger snails are constantly being removed from the population by waves at those sites, survivorship and mean snail size will both decrease. In contrast, larger snails are more resistant to desiccation (Poznanska, Kakareko, Gulanicz, Jermacz, & Kobak, 2015) and less susceptible to fish predators (Byers, Malek, Quevillon, Altman, & Keogh, 2015). This size advantage may explain the positive correlation between size and survival observed at Nudibranch Point. As with trematodes, a direct connection between snail survival and size is not clear. Instead, environmental factors within each site and snail size appear to interact to determine survival probabilities.

Independent of snail size, periwinkle survival was higher on mainland than on islet sites. Several environmental factors differ between the two site types that can explain our results, including the following: (1) Resource availability. Some species of *Fucus* are used as a primary food resource by littorinid snails from intertidal habitats (Granovitch & Maximovich, 2013; Kozminsky, 2013). Mainland sites have more macroalgae cover of *Fucus* sp. (Mónica Ayala‐Díaz, personal observation), which plays an important role in snail survival (Chapman, 1997). (2) Population density. Sites on the mainland have larger surface area than sites on the islets, reducing population density, and intraspecific competition, and thereby increasing survival probability (Kozminsky, 2013). A combined effect of food availability and population density might explain the higher survival which we observed in the laboratory compared to the field. Snail samples in the laboratory were maintained at constant density with unlimited food availability. However, these conditions are unlikely to occur in nature where recruitment and food availability are constantly shifting. (3) Terrain. The rocky intertidal zone on the mainland sites has minimal slope and tide pools in close proximity to each other, facilitating snail movement to suitable microhabitats, and thereby increasing survival probabilities. In contrast, islet sites have steep slopes and irregular rocks with tide pools spread farther apart, potentially impeding snail relocation and microhabitat selection, leading to reduced survival rates. (4) Shelter. Crevices in rocks protect snails against temperature, desiccation, wave exposure, and predation (Behrens Yamada & Boulding, 1996; Boulding & Van Alstyne, 1993; Catesby & McKillup, 1998; Kovach & Tallmon, 2010). The rocky intertidal in the islets has more rock crevices, likely increasing survival of smaller snails.

Survival and recapture estimates using both MARK and Bayesian models were very similar. The Bayesian approach provided two advantages. First, we were able to incorporate individual snail size as a covariate and thus detect effects of snail size on survival and recapture rates. Second, we were able to use information on known mortality, while the analysis in MARK required us to remove capture history information of individuals found dead. Deleting data of known mortality can lead to survival overestimation; thus, WinBUGS provides more conservative survival estimates. Survival estimates from Prasiola Point and Ross and Wizard islets were slightly higher from MARK than from WinBUGS, in keeping with survival overestimates using ML and supporting the advantages of adding information from dead recoveries while estimating survival.

## 5 Conclusion

We found significant differences in snail survival among sites, but underlying causes remain unclear. Trematode species found in this study do not appear to have a direct negative effect on health of *L. sitkana*; infected snails kept in the laboratory have higher survival than snails studied in the field. Thus, factors other than trematode infection are likely to be more important for survival of *L. sitkana* in our study system. Our results suggest that an interaction between snail size and predator presence and/or wave exposure may lead to differences in snail survival. Other environmental factors such as resource availability, population density, and refuge availability may also affect snail survival in our study sites.

## Conflict of Interest

None declared.

## Appendix 1

### 1.1

**Table A1:** Akaike information criterion (AIC) rankings of all models compared in this study to determine if trematode presence has an effect on survival and recapture estimates of *Littorina sitkana*

Rank	Model	QAICc	ΔQAICc	No. Parameters	Deviance
1	Φ(site × time) p(site × time)	26159.99	0.00	60	1271.84
2	Φ(site × time) p(site + time)	26262.82	102.83	42	1410.85
3	Φ(site × Trem‐long term) p(site × time)	26283.27	123.28	39	1437.32
4	Φ(site + Trem‐long term) p(site × time)	26341.55	181.56	37	1499.62
5	Φ(site + time) p(site × time)	26344.12	184.13	42	1492.15
6	Φ(site × Trem‐immediate) p(site × time)	26346.69	186.70	40	1498.74
7	Φ(site + Trem‐immediate) p(site × time)	26377.75	217.76	37	1535.81
8	Φ(site × Trem‐long term) p(site + time)	26442.85	282.85	19	1637.01
9	Φ(site × Trem‐immediate) p(site + time)	26453.48	293.49	19	1647.65
10	Φ(site + time) p(site + time)	26468.88	308.89	22	1657.04
11	Φ(site + Trem‐long term) p(site + time)	26469.62	309.63	16	1669.80
12	Φ(site + Trem‐immediate) p(site + time)	26496.79	336.80	16	1696.97
13	Φ(site × Trem‐long term) p(site × Trem‐long term)	26503.55	343.55	16	1703.72
14	Φ(site × Trem‐long term) p(site + Trem‐long term)	26551.11	391.12	13	1757.30
15	Φ(site + Trem‐long term) p(site × Trem‐long term)	26556.61	396.62	13	1762.79
16	Φ(site + Trem‐long term) p(site + Trem‐long term)	26594.70	434.71	10	1806.89
17	Φ(site + time) p(site)	26640.43	480.44	15	1842.61
18	Φ(site × Trem‐immediate) p(site × Trem‐immediate)	26733.53	573.54	16	1933.71
19	Φ(site × Trem‐immediate) p(site + Trem‐immediate)	26771.73	611.73	13	1977.91
20	Φ(site + Trem‐immediate) p(site × Trem‐immediate)	26783.35	623.36	13	1989.53
21	Φ(Trem‐long term) p(site + Trem‐long term)	26831.61	671.62	7	2049.81
22	Φ(site + Trem‐immediate) p(site + Trem‐immediate)	26838.85	678.86	10	2051.04
23	Φ(site) p(site)	26847.46	687.47	8	2063.66
24	Φ(Trem‐immediate) p(site + Trem‐immediate)	27091.53	931.54	7	2309.73
25	Φ(site + Trem‐long term) p(Trem‐long term)	27383.52	1223.53	7	2601.72
26	Φ(site + Trem‐immediate) p(Trem‐immediate)	27558.80	1398.81	7	2776.99
27	Φ(Trem‐long term) p(Trem‐long term)	27721.41	1561.42	4	2945.61
28	Φ(Trem‐immediate) p(Trem‐immediate)	27889.83	1729.84	4	3114.03
